# B cell fate mapping reveals their contribution to the memory immune response against helminths

**DOI:** 10.3389/fimmu.2022.1016142

**Published:** 2022-11-24

**Authors:** Paul Haase, Simon Schäfer, Roman G. Gerlach, Thomas H. Winkler, David Voehringer

**Affiliations:** ^1^ Department of Infection Biology, Universitätsklinikum Erlangen, Erlangen, Germany; ^2^ Department of Genetics, Faculty of Sciences, Friedrich-Alexander Universität Erlangen-Nürnberg (FAU), Erlangen, Germany; ^3^ Institute of Clinical Microbiology, Immunology and Hygiene, Universitätsklinikum Erlangen, Erlangen, Germany; ^4^ Faculty of Medicine, Friedrich-Alexander Universität Erlangen-Nürnberg (FAU), Erlangen, Germany

**Keywords:** germinal center, plasma cells, fate mapping, helminths, memory responses

## Abstract

An estimated quarter of the human world population is infected with gastrointestinal helminths causing major socioeconomic problems in endemic countries. A better understanding of humoral immune responses against helminths is urgently needed to develop effective vaccination strategies. Here, we used a fate mapping (FM) approach to mark germinal center (GC) B cells and their developmental fates by induced expression of a fluorescent protein during infection of mice with the helminth *Nippostrongylus brasiliensis*. We could show that FM^+^ cells persist weeks after clearance of the primary infection mainly as CD80^+^CD73^+^PD-L2^+^ memory B cells. A secondary infection elicited expansion of helminth-specific memory B cells and plasma cells (PCs). Adoptive transfers and analysis of somatic mutations in immunoglobulin genes further revealed that FM^+^ B cells rapidly convert to PCs rather than participating again in a GC reaction. These results provide new insights in the population dynamics of the humoral immune response against helminths.

## Introduction

Currently about 1.5 billion people worldwide are affected by soil-transmitted gastrointestinal helminths. These pathogens are large and complex multicellular organisms which express multiple antigens both on their body surface and in form of secreted molecules. Helminths often elicit a type 2 immune response which is also characteristic for an allergic immune response and initiated by the cytokine alarmins IL-33, IL-25 and TSLP among other factors (1, 2). This leads subsequently to an increase of Th2 cells and type 2 innate lymphoid cells (ILC2s), secretion of cytokines such as IL-4, IL-5 and IL-13, accumulation of basophils and eosinophils in tissues, activation of goblet cells, mast cells and smooth muscle cells, differentiation of alternatively activated macrophages and high serum levels of IgE and IgG1. Nonetheless, there are several important differences between an allergic type 2 response and the type 2 response against parasites (3). For example, we still do not know the exact mechanisms that regulate differences in the generation, maintenance and recall of the humoral immune response observed during helminth infection versus allergic inflammation (4–6). Several studies demonstrated that B cells and antibodies contribute to protective immunity against gastrointestinal helminths in various ways (7–10). However, the formation and reactivation of memory B cells and antibody-secreting plasma cells (PCs) during primary and secondary helminth infection remains poorly defined.

Several recent studies showed that memory B cells are generally quite heterogeneous and that their functions can be instructed during their ontogeny (11–13). Some reports demonstrated that memory B cells produced early on in the immune response have lower affinity and primarily express IgM and IgD but can re-enter germinal centers (GCs) and create new memory B cells (14–16). In contrast, the memory B cells produced later during the immune response are considered to be the ones with higher affinity of the B cell receptor and rapid conversion to PCs upon antigen challenge to provide fast and highly specific protection (11, 14–17). Nonetheless, it is still debated if GC re-entry of memory B cells is a relevant process or whether their function is mainly to provide immediate protection *via* differentiation to PCs and antibody secretion (13, 18–21). Furthermore, many studies are based on immunization protocols with haptens such as nitrophenol (NP) that allow identification and fate-mapping of B cells specific for a single antigen but does not really reflect the population dynamics of a polyclonal B cell response directed against a complex pathogen with many different antigens in a natural infection model.

Hence, we aimed at investigating the fate of B cells activated during an immune response against helminths using the *Nippostrongylus brasiliensis* infection model of mice. In brief, infective L3 stage larvae are injected subcutaneously and migrate first to the lung and then to the small intestine were they develop to adults and get expelled about 10 days after infection (22). A secondary infection is cleared much more rapidly by several mechanisms including the antibody-mediated activation of basophils (9, 23, 24). We previously showed that B cell-intrinsic expression of the IL-4/IL-13-activated transcription factor STAT6 was required for GC formation during *N. brasiliensis* infection or immunization with OVA/alum or sheep red blood cells (25). In addition, we and others demonstrated that the IgE memory response was preserved on the level of IgG1^+^ B cells in the mesenteric lymph nodes and the bone marrow (26–28). Adoptively transferred memory IgG1^+^ B cells rapidly converted to IgE-secreting plasma cells upon *in vivo* re-challenge (26, 28).

In the present study we used an inducible Cre system to label B cells participating in the immune response against *N. brasiliensis*. These Aid-CreERT2 x tdTomato mice express a tamoxifen-inducible Cre recombinase from the *Aicda* locus enabling temporal Cre activation which in turn removes the stop cassette in front of the tdTomato cassette in the *Rosa26* locus to permanently label those cells and their offspring with the fluorescent protein tdTomato (14). Using this fate mapping approach, we first infected mice with *N. brasiliensis* followed by a single i.p. injection of tamoxifen on day seven after infection. We analyzed the mice on day 10 after primary infection, in the memory phase about 3 weeks after worm expulsion, and on day 10 after a challenge infection. Using flow cytometry, adoptive transfers, ELISA and immunoglobulin (Ig) repertoire analysis we could characterize the fate of GC-derived B cells elicited during primary helminth infection.

## Materials and methods

### Mouse models

Aid-CreERT2^ki/ki^ mice (14) were crossed with R26-LSL-tdTomato^fl/fl^ (Ai14) mice (29) to generate Aid-CreERT2^ki/wt^ x R26-LSL-tdTomato^fl/fl^ mice (Aid-CreERT2 x tdTomato). Ly5.1_ IgH^a^ mice were generated by crossing IgH^a^-B6 mice (B6.Cg-Gpi1a Thy1a Igha/J, available from the Jackson Laboratory) with Ly5.1-B6 mice (B6.SJL-Ptprca Pepcb/BoyJ, available from the Jackson Laboratory). All mice were on C57BL/6 background and housed according to the institutional guidelines. Both male and female mice were used at the age of 8-12 weeks.

### Infection and tamoxifen treatment

Mice received 500 L3 stage larvae of *N. brasiliensis* s.c. in the back whereas control mice received equal volumes of saline solution. Seven days later mice received a single i.p. injection of 1 mg tamoxifen (Sigma-Aldrich) dissolved in Miglyol 812 (Caesar & Loretz). For challenge infection mice received same number of larvae as for primary infection while control mice received 500 L3 larvae for the first time. Mice were analyzed 10 days after primary or challenge infection or during the memory phase at least 28 days after primary infection.

### Flow cytometry

Flow cytometric analysis was performed in accordance to published guidelines (30). Single cell suspensions of bone marrow (BM), mesenteric lymph nodes (mes-LN) and mediastinal lymph nodes (med-LN) were generated by mechanical disruption. Erythrocytes in the BM were lysed with ACK-buffer (0.15M NH_4_Cl, 1mM KHO_3_, 0.1mM Na_2_EDTA) and all cells resuspended in FACS buffer (PBS, 2% FCS, 1 mg/mL NaN_3_). For staining of IgE-expressing cells cytophilic IgE was efficiently removed by short treatment with acetate buffer as described (26). Fc receptors were blocked with anti-mouse CD16/CD32 mAb (BioXcell) for 5 min at RT and the primary antibodies were incubated for 25 min at 4°C in the dark (for list of antibodies used refer to **Supplementary Table S1**).

As secondary staining streptavidin conjugated to BUV 395 was added and the cells were incubated for 20 min at 4°C in the dark. For PCs and GC B cells during primary and secondary infection IgA, IgE and IgG1 were instead intracellularly stained using Cytofix/Cytoperm™ (BD Bioscience) and Phosflow™ Perm/Wash Buffer I (BD Bioscience). Cells were analyzed on a BD LSR-Fortessa instrument (BD Bioscience).

### Adoptive transfer

Cells were isolated from mes-LN and BM on day 28 or later after infection and were purified with the MojoSort™ Mouse Pan B Cell Isolation Kit II (BioLegend). 2-3x10^6^ cells were transferred i.v. into Ly5.1_IgH^a^ recipient mice which had been infected with *N. brasiliensis* three days before. Mice were analyzed on day 10 after infection. BM, mes-LN and med-LN samples were taken for flow cytometric analysis and serum was used for IgE ELISA.

### IgE ELISA

Plates were coated overnight at 4°C with purified rat anti-mouse IgE (BD Bioscience) followed by blocking with 3% BSA. Duplicates for each samples were prepared. Next, plates were incubated at RT with serial dilutions of IgE^a^ or IgE^b^ allotype standard (BD Bioscience) and serum. Afterwards detection antibodies for either total IgE (Southern Biotech), IgE^a^ (BD Bioscience) or IgE^b^ (BD Bioscience) were added. Additional incubation with AP-coupled streptavidin (Southern Biotech) was required for the allotype-specific ELISAs. Freshly prepared PNPP-substrate in AP-buffer (100mM Tris-Cl; 100mM NaCl; 5mM MgCl2; 0.01% NaN3; pH:9.5) was added and absorption was measured at 405 nm on a Multiskan FC photometer (Thermo Fisher). Concentration was calculated using the mean absorption of the respective duplicates using the respective standards. The mean of three to four time points per sample was used to calculate the final concentration. Controls for the specificity of IgE^a^ and IgE^b^ ELISAs are shown in **Figure S4E**.

### Sequencing

Cells were isolated from mes-LN and BM on day 28 or later after infection and on day 10 after transfer and B cells were purified with the MojoSort™ Mouse Pan B Cell Isolation Kit II (BioLegend). For cells after transfer Fc receptors were blocked with anti-mouse CD16/CD32 mAb and the cells stained at 4°C for 30 min for CD45.2 (Biolegend). Cells were sorted for tdTomato^+^ and tdTomato^-^ before transfer and CD45.2^+^ tdTomato^+^ or CD45.2^+^ Tomato^-^ cells after transfer using the S3 sorter (Bio-Rad). The cells were sorted into 1 ml FCS, pelleted and immediately lysed with RLT-buffer (RNeasy-Kit; Qiagen). The lysate was stored at -80°C until RNA isolation. RNA was isolated using the RNeasy micro kit (Qiagen). The samples were prepared for sequencing and analyzed according to the procedures described in (31).Samples were sequenced in an illumina MiSeq machine using the MiSeq Reagent Kit v3 (600-cycle).

### Quantification and statistical analysis

Data are presented as mean values with standard error of the mean (SEM). One-way ANOVA followed by Holm-Sidak test was used for multiple comparisons. Student’s t-testwas used for pair-wise comparisons. Data was log-transformed before analysis of cell count data. Statistical analysis was performed *via* SigmaPlot (Systat Software) and a p-value < 0.05 was considered significant.

## Results

### Population dynamics of fate-mapped GC B cells and plasma cells after *N. brasiliensis* infection

**Figure 1:**
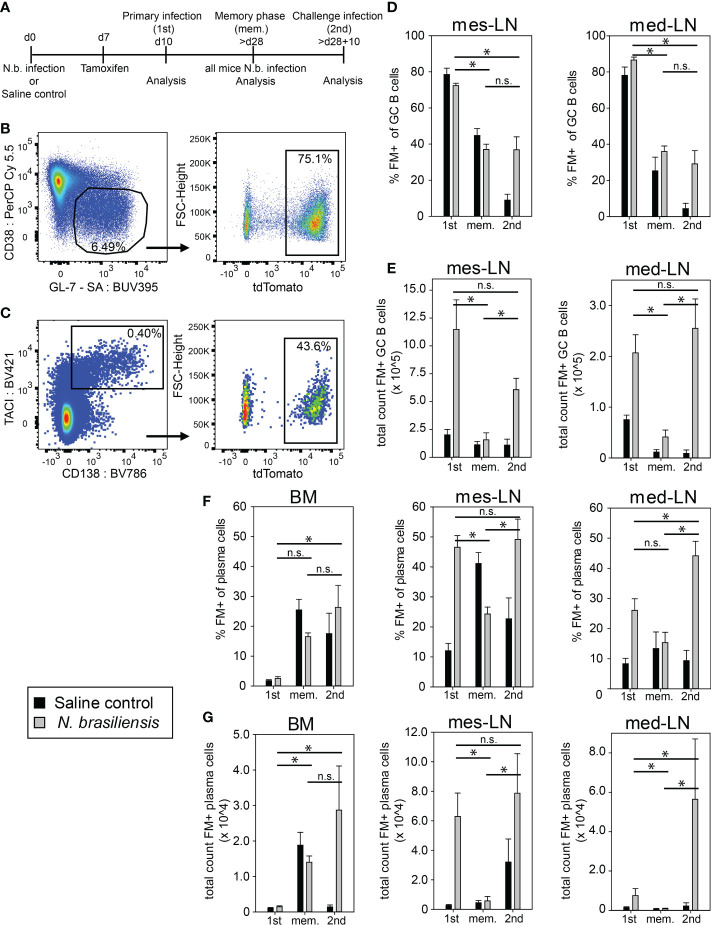
Population dynamics of fate-mapped GC B cells and plasma cells after *N. brasiliensis* infection. **(A)** Outline of experimental setup. **(B, C)** Representative dot plots of FM^+^ (tdTomato^+^) GC B cells **(B)** or plasma cells **(C)** on day 10 after primary infection (gating strategy **Figure S1A**). **(D, E)** FM^+^ GC B cells in percentage **(D)** or total count **(E)** during primary infection (1^st^), in the memory phase (mem.), and after challenge infection (2^nd^). **(F, G)** FM^+^ PCs in percentage **(F)** or total count **(G)** during 1^st^ infection, in the memory phase (mem.), and after 2^nd^ infection. Data shows mean with SEM from 2-3 experiment with 4-8 mice per group. Significance was tested with ANOVA followed by Holm-Sidak test. Data of total cell count was log-transformed before testing for significance. * (p < 0.05); n.s. (not significant).

To follow the fate of helminth-elicited GC B cells, we infected Aid-CreERT2 x tdTomato mice with *N. brasiliensis* and injected 1mg tamoxifen on day 7. Mice were analyzed on day 10 after primary infection, during the memory phase after about 28 days, and 10 days after challenge infection. As control we used littermates who received saline solution instead of a primary infection but were otherwise treated the same as the experimental group (**Figure 1A**). The frequency of fate map positive (FM^+^) GC B cells among all GC B cells (B220^+^CD38^lo^GL-7^+^) in mediastinal (med) and mesenteric (mes) lymph nodes on day 10 during 1^st^ infection was around 80% (**Figures 1B, D**). We observed a similar frequency of FM^+^ GC B cells in the control group indicating that GC B cells existing under steady-state conditions in our SPF facility are also efficiently labeled (**Figure 1D**). However, the total number of FM^+^ GC B cells in infected mice was 3-5 fold higher as compared to the saline control group at this timepoint (**Figure 1E**). Control experiments with tamoxifen administration to Cre-negative mice or infections of Cre-positive mice without tamoxifen administration revealed very low background labeling (**Figure S1F**). In the memory phase, the frequency of FM^+^ GC B cells of both groups dropped to 30-40% in both LNs and the total number of FM^+^ GC B cells of the infected group declined to a level comparable to the saline control group (**Figures 1D, E**). Interestingly, after 2^nd^ infection, FM^+^ GC B cells in the infected group remained at 30-40% but total numbers increased again suggesting that they partially participate in a secondary GC response but that also new (FM^-^) B cells constitute at least half of the total GC B cell population (**Figures 1D, E**). Importantly, the saline group showed only 5-10% FM^+^ GC B cells and no increase in total numbers upon *N. brasiliensis* infection (**Figures 1D, E**). Therefore, we conclude that the vast majority of FM^+^ GC B cells after 2^nd^ infection are indeed *N. brasiliensis*-specific B cells.

Next, we investigated the generation of FM^+^ PCs. After 1^st^ infection we observed a 3-4 fold higher frequency of FM^+^ PCs among all PCs (B220^-^CD138^+^TACI^+^) and about 10-20 times more FM^+^ PCs in lymph nodes of the experimental group as compared to the saline control group whereas only few FM^+^ PCs could be found in the bone marrow (BM) (**Figures 1C, F, G**). During the memory phase FM^+^ PCs were mainly found in the BM where they accounted for about 20% of all PCs in both groups of mice. After 2^nd^ infection FM^+^ PCs frequencies and total numbers were maintained in BM and increased again in lymph nodes of the experimental group but not in the saline controls (**Figures 1F, G**). We further analyzed one set of mice where we performed 2^nd^ infection at 8 months after 1^st^ infection and obtained about 2-fold less FM^+^ GC B cells and PCs in mes-LN as compared to the results at 4 weeks after 1^st^ infection (**Figure S1D, E**). This indicates that the vast majority of FM^+^ PCs in both lymph nodes on day 10 after 1^st^ or 2^nd^ infection were indeed generated in response to the infection while in the BM this is only the case after 2^nd^ infection.

### Expression of Ig isotypes among FM^+^ cells after 1^st^ and 2^nd^ infection

**Figure 2:**
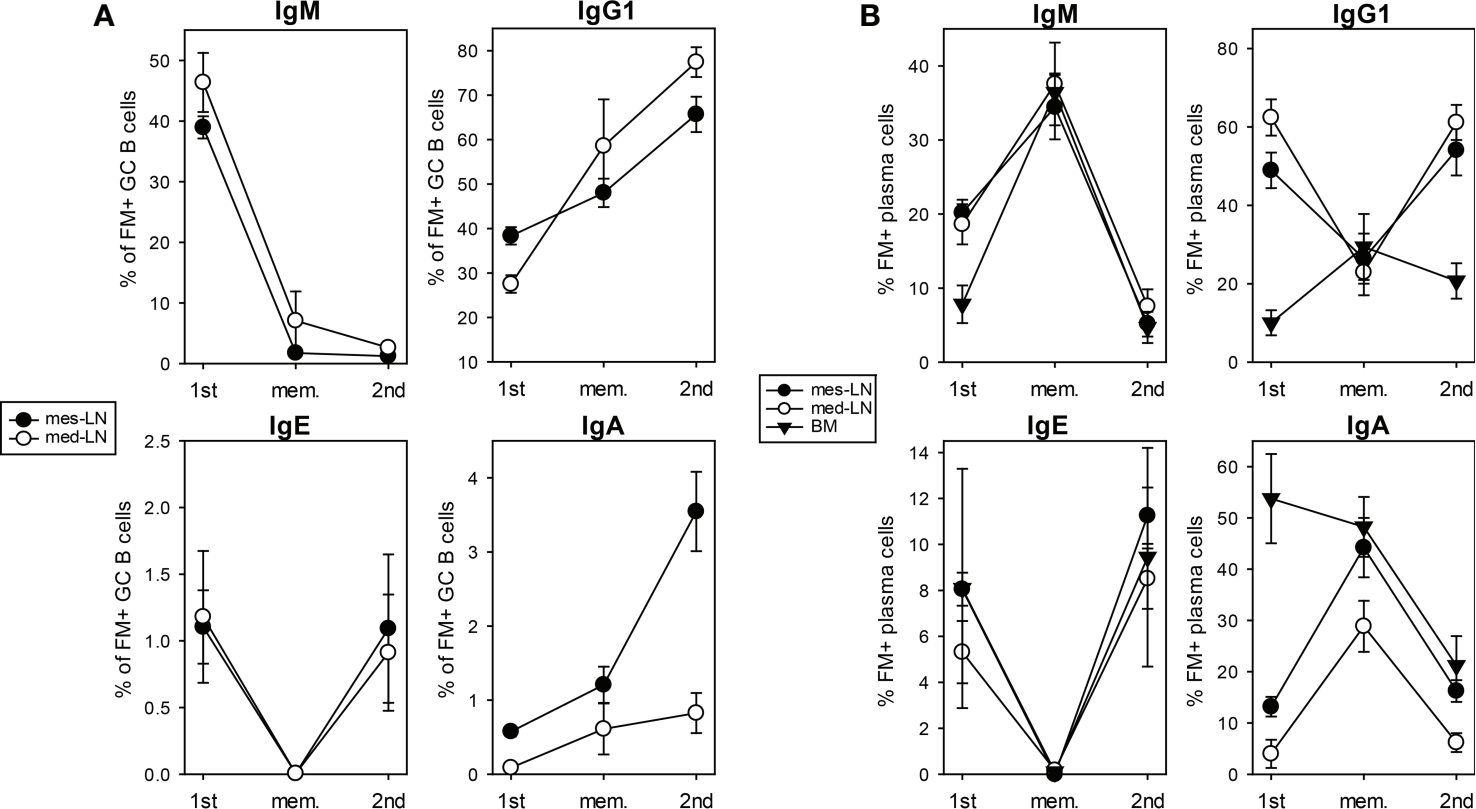
Expression of Ig isotypes among FM^+^ cells after 1^st^ and 2^nd^ infection. Percentage of FM^+^ GC B cells **(A)** or PCs **(B)** that express IgM, IgG1, IgE or IgA (gating strategy Figure S1B, C) on day 10 primary infection (1^st^) infection, in the memory phase (mem.) and on day 10 after challenge infection (2^nd^), in mes-LN, med-LN or BM. Data shows mean with SEM from 1-3 experiments with 4-8 mice per group.

After we had investigated the population dynamics of FM^+^ GC B cells and PCs we further analyzed the expression of different Ig isotypes in these cells. In both lymph nodes the population of IgM^+^ cells among all FM^+^ GC B cells decreased from about 40% after 1^st^ infection to less than 10% in the memory phase and after 2^nd^ infection, while IgG1^+^ cells increased from 30% after 1^st^ infection to 50% in the memory phase and about 70% after 2^nd^ infection (**Figure 2A**; **S1B, C**). About 1% of all FM^+^ GC B cells were IgE^+^ in both lymph nodes after 1^st^ infection. These cells disappeared during the memory phase and reappeared with the same frequency after 2^nd^ infection (**Figure 2A**). IgA^+^ GC B cells increased in the mes-LN (from 0.5% to 3.5%) during 2^nd^ infection while they remained at a low level in the med-LN (**Figure 2A**).

About 20% of all FM^+^ PCs in both lymph nodes and 10% of all FM^+^ PCs in the BM were IgM^+^ after 1^st^ infection (**Figure 2B**). This value increased to 35% in all three organs during the memory phase suggesting a survival advantage and decreased equally to below 10% after 2^nd^ infection. In both lymph nodes the IgG1^+^ population of FM^+^ PCs decreased from 50-60% after 1^st^ infection to 25% in the memory phase and increased again to around 50% after 2^nd^ infection. In the BM 10% of FM^+^ PCs were IgG1^+^ after 1^st^ infection, which slightly increases during the memory phase to 25% and dropped to 20% after 2^nd^ infection. For IgE^+^ cells within the FM^+^ PCs we detected in all three organs a frequency of around 6% after 1^st^ infection, a drop to almost zero during the memory phase and an increase to 10% after 2^nd^ infection. The frequency of IgA^+^ cells within FM^+^ PCs in the BM decreased from 50% after 1^st^ infection to 20% after 2^nd^ infection. In contrast, in the lymph nodes we found around 5-15% IgA^+^ cells within the FM^+^ PC population after 1^st^ infection, an increase to 40% in the memory phase and a decline to 10% after 2^nd^ infection.

We further analyzed the frequency of FM^+^ cells within GC B cells and PCs expressing different Ig isotypes. On day 10 after 1^st^ infection about 70-90% of IgM^+^, IgG1^+^, IgE^+^ or IgA^+^ GC B cells were FM^+^ (**Figure S2A**). This frequency went down to 40% in the memory phase and remained there after 2^nd^ infection. For the PC population we found that after 1^st^ infection 60-80% of IgG1^+^ or IgE^+^ PCs were FM^+^ in both LNs but not in the BM. Also here, the frequency of FM^+^ cells went down in the memory phase but raised again after 2^nd^ infection for the IgE^+^ PC population and we then found FM^+^ IgE^+^ PCs in the BM (**Figure S2B**).

These results demonstrate the highly dynamic alterations of different GC B cell and PC populations expressing distinct Ig isotypes.

### Expansion of memory B cell subsets after 2^nd^ infection with *Nippostrongylus brasiliensis*

**Figure 3:**
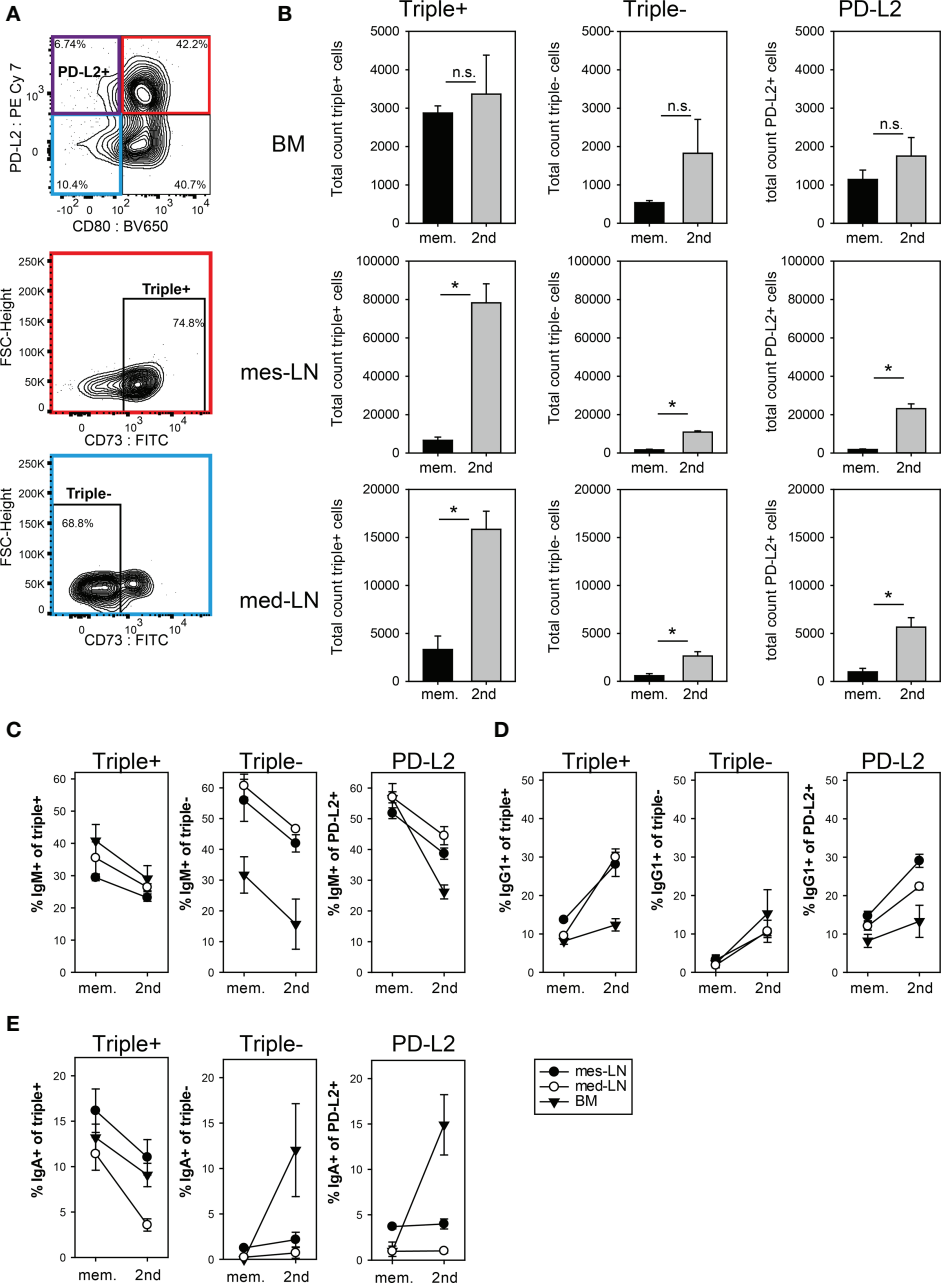
Expansion of memory B cell subsets after 2^nd^ infection with *N. brasiliensis*. **(A)** Gating strategy for the three memory subpopulations PD-L2^+^ (violet), triple^+^ (red) and triple^-^ (blue). Cells from the mes-LN gated for FM^+^ B220^+^ CD38^+^ GL7^-^ CD138^-^ (refer to Figure S3A). **(B)** Total count of FM^+^ memory B cells in the three subpopulations during the memory phase (mem.) and 10 days after 2^nd^ infection. **(C–E)** Percentage of FM^+^ memory B cell subpopulations that express IgM **(C)**, IgG1 **(D)** or IgA **(E)** during the memory phase (mem.) and 10 days after 2^nd^ infection (gating Figure S3B) in mes-LN, med-LN or BM. Data shows mean with SEM from 1-2 experiments with 4-5 mice per group. Student’s t-test was performed after log-transforming the data. * (p < 0.05); n.s. (not significant).

To further investigate the composition of the memory B cell population, we analyzed mice at least 28 days after initial infection and 10 days after a challenge infection using the fate-map tdTomato signal as identifier of memory B cells in addition to the published markers CD73, CD80 and PD-L2 to differentiate between subsets of memory B cells (32). We focused on three distinct FM^+^ memory B cell populations, the triple^+^ cells (CD73^+^, CD80^+^, PD-L2^+^), triple^-^ cells (CD73^-^, CD80^-^, PD-L2^-^) and PD-L2^+^ cells (PD-L2^+^, CD80^-^ CD73^variable^) (**Figure 3A**, **S3**). This definition of memory B cell subsets is based on results from a hapten immunization model, where the triple^+^ population was reported to correspond to the most memory-like cells while the triple^-^ population indicated more naïve-like cells (32). The triple^+^ population dominated in the BM and both LNs in the memory phase before challenge infection. After challenge infection, the triple^+^ cells increased 5-10 fold in both LNs but not in the BM. The triple^-^ and the PD-L2 populations also significantly increased in the LNs after challenge infection but remained minor populations (**Figure 3B**).

Interestingly, in the memory phase about 30-40% of the triple^+^ population in all three organs expressed IgM and only 10% expressed IgG1 (**Figure 3C**). After challenge infection, the IgM frequencies dropped to 25-30% and IgG1 frequencies increased to about 30% in LNs but not in the BM. Similar results were obtained for the triple^-^ and PD-L2 populations, although they had even higher IgM frequencies with exception of the triple^-^ BM population (**Figures 3C, D**). For IgA the frequency dropped from 10-16% to 5%-12% for the triple^+^ population whereas triple^-^ and PD-L2 IgA cells remained at around 3% in the mes-LN and med-LN and only in the BM did we observe an increase of triple^-^ and PD-L2 IgA memory B cells (**Figure 3E**).

### Transferred FM^+^ cells preferentially differentiate to PCs.

**Figure 4:**
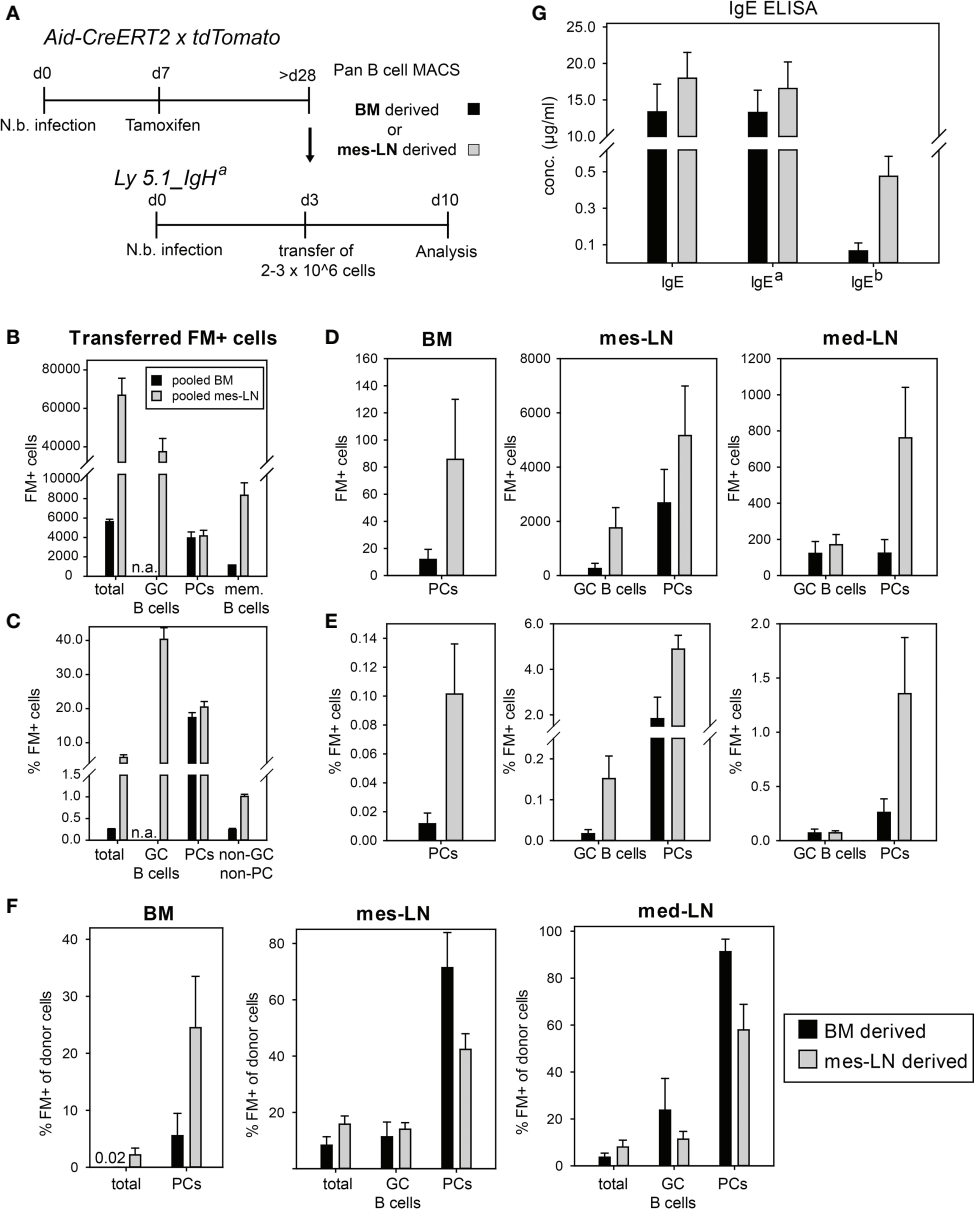
Transferred FM^+^ cells preferentially differentiate to PCs. **(A)** Outline of experimental setup. **(B)** Average number of FM^+^ populations in pooled cells transferred per mouse. **(C)** Percentage of FM^+^ cells in indicated donor cell populations. **(D, E)** Total count **(D)** or percentage **(E)** of FM^+^ cells in GC B cells or PCs after transfer in BM, mes-LN or med-LN. **(F)** Percentage of FM^+^ cells from donor cells after transfer in BM, mes-LN or med-LN. **(G)** Concentrations of total IgE, IgE^a^ and IgE^b^ allotypes in the serum of recipient mice on day 10 of infection. Data shows mean with SEM from 2 experiments with 6 mice per group.

To further determine whether memory B cells in BM or mes-LNs behave differently after re-challenge, we performed adoptive transfer experiments. Therefore, we first infected Aid-CreERT2 x tdTomato mice with *N. brasiliensis*, labelled GC B cells on day 7 by tamoxifen injection and isolated total B cells from either the mes-LN or the BM of several mice. These two populations were first analyzed for expression of the FM signal, then transferred separately into congenic Ly5.1_IgH^a^ recipient mice which had been infected with *N. brasiliensis* three days before and analyzed 7 days later (**Figure 4A**). This setup allows the detection of donor-derived cells (Ly5.2^+^) and antibodies produced by donor-derived PCs (IgH^b^). Each recipient received 2-3x10^6^ cells which contained about 6,000 (BM) or 60,000 (mes-LN) FM^+^ cells with approximately 4,000 of those cells being PCs in both samples and 1,000 (BM) or 8,000 (mes-LN) memory B cells (FM^+^ B220^+^ CD38^hi^ GL-7^lo^ CD138^-^). The largest population (about 40,000 cells) of the transferred FM^+^ B cells from the mes-LN node showed a GC B cell phenotype (**Figure 4B**). The frequency of FM^+^ cells was 40% in GC B cells and 20% in PCs (**Figure 4C**). GC B cells and PCs in the transferred donor cells are generally considered to have a rather poor reconstitution capacity so that the majority of donor-derived FM^+^ cells in the host are most likely derived from memory B cells.

After the transfer we observed only few FM^+^ PCs in the BM (20 and 80 cells for BM- and mes-LN-derived cells, respectively). Contrarily, in the mes-LN of the recipient mice we detected around 200 FM^+^ cells with a GC B cell phenotype (CD38^lo^GL-7^hi^) from the BM donor population, 2,000 FM^+^ GC B cells derived from the mes-LN donor population and 2,000 or 6,000 FM^+^ PCs, respectively. In the med-LNs of the recipient mice we found around 200 FM^+^ GC B cells for both BM and mes-LN derived cells and detected 200 and 800 FM^+^ PCs, respectively (**Figure 4D**). These numbers correspond to less than 0.2% of total GC B cells but up to 5% of the total PCs in the mes-LN and med-LN and 0.1-0.2% of PCs in the BM (**Figure 4E**).

Since we transferred not only FM^+^ cells but also FM^-^ cells we next determined the frequency of FM^+^ cells within the total donor-derived B cell and PC populations in BM and mes-LN of the recipient mice. In the mes-LN and med-LN we observed that the frequency of FM^+^ PCs increased from 20% before transfer to 80-90% of the BM-derived donor PCs and 40-60% of the mes-LN-derived PCs. In contrast, in the BM of the recipient mice the frequency of FM^+^ PCs within the donor cells was only 10% for BM-derived cells and 25% for mes-LN derived cells (**Figure 4F**). For donor GC B cells we observed that the frequency of FM^+^ cells decreased from 40% before transfer to 10-20% after transfer and rechallenge (**Figures 4C, F**).

Finally, we analyzed whether the donor-derived PCs participated in the immune response against *N. brasiliensis* of the recipient mice. For this we determined serum IgE levels and distinguished between donor and recipient IgE *via* the allotype of the Ig constant region with the host PCs secreting IgE^a^ and the donor PCs secreting IgE^b^. As expected, most of the serum IgE was produced by host PCs. However, we also detected donor-derived IgE which constituted about 1% of total IgE in case of the BM transfer and 5% in case of mes-LN transfer (**Figure 4G**). This result corresponds to the difference in total FM^+^ PC numbers generated from the two different donor populations (**Figure 4D**) and the frequency of PCs in the lymph nodes (**Figure 4E**).

### Immunoglobulin genes of most FM^+^ cells do not acquire more somatic mutations after transfer and re-challenge

**Figure 5:**
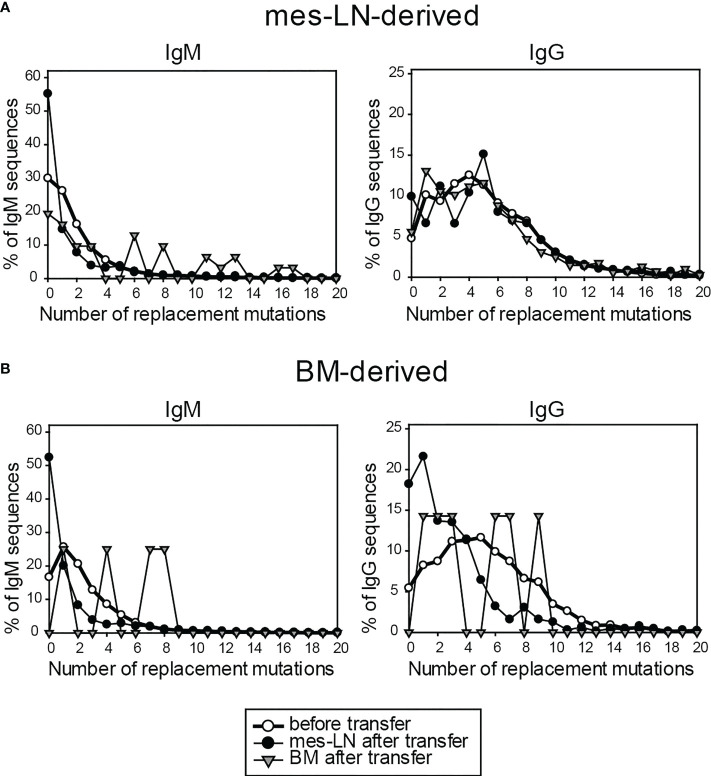
Immunoglobulin genes of FM^+^ cells do not acquire more somatic mutations after transfer and re-challenge. Frequency of replacement mutations of FM^+^ cells on day 10 after transfer for IgM and IgG, compared to cells before transfer. Cells derived from either mes-LN **(A)** or BM **(B)** of the donor mice. Data shows pooled samples of 3 mice per group.

Finally, we analyzed the number of somatic mutations in genes of the immunoglobulin heavy chains of different isotypes from FM^+^ cells in the memory phase before and after transfer and re-challenge by next-generation sequencing. When we compared the sequences of mes-LN-derived cells before transfer with sequences from donor cells isolated from mes-LN or BM after re-challenge, we observed no obvious increase of replacement mutations for IgM, IgG and IgE (**Figure 5A**). Furthermore, when we analyzed sequences of cells derived from the BM donor population, the frequency of highly mutated sequences even decreased after transfer and re-challenge for IgM and IgG whereas IgE sequences were infrequent and not detectable in the BM after transfer (**Figure 5B**). The caveat of this analysis is the fact that most sequences are derived from PCs which express much more immunoglobulin mRNA as compared to memory B cells or GC B cells. Therefore, we cannot exclude the possibility that some IgM^+^ memory B cells participated in a GC response and became IgG1^+^ memory B cells with more somatic mutations.

## Discussion

The fate of GC-derived B cells after helminth infection is poorly understood. Here, we used a fate-mapping approach to address this point in the context of *N. brasiliensis* infection, a well-established model for human hookworm infections. We show that a single injection of tamoxifen in AIDCreERT2 x tdTomato mice leads to efficient labeling of GC-B cells and allows tracking of their cellular fates during the memory phase and upon re-challenge. This fate-mapping approach has been used before in other settings and is especially usefull when specific antigens of the humoral immune response are not known or polyconal B cell responses are being investigated (14, 33). However, commensals and environmental antigens constantly activate B cells in mucosal tissues causing a certain background of FM^+^ cells that are not specific for the pathogen used for infection. This was reflected by the fact that both saline control and *N. brasiliensis* infected mice showed similar frequencies of FM^+^ GC B cells after the first infection and during the memory phase. However, the total number of FM^+^ GC B cells and PCs was much larger in *N. brasiliensis*-infected mice as compared to saline controls. This difference disappeared in the memory phase due to the decline of the helminth-elicited GC response. Some FM^+^ cells could also be derived from activation of AID in extrafollicular B cells giving rise to short-lived plasmablasts and memory B cells (34). In fact, it has been shown that memory B cells with few somatic mutations can arise independently of GCs (35, 36). Importantly, the secondary expansion of the FM^+^ GC B cell and PC populations after challenge infection was only observed in the group of mice that had been infected with *N. brasiliensis* during the fate-map labeling and persisted up to 8 months after the initial infection with no significant reduction of FM^+^ cells after challenge infection (**Figure S1D**). This demonstrates the antigen-specific expansion of memory B cell and PC populations. We could not distinguish whether the memory B cells were derived from GCs or extrafollicular space. However, the adoptive transfer and sequencing experiment indicates that early memory B cells might indeed be relevant in this model since only few somatic mutations were detected in FM^+^ cells after transfer and rechallenge. This point should be addressed in future studies e.g. by using anti-ICOS blocking antibodies (35). The disappearance of IgM^+^ cells and increase of IgG1^+^ and IgE^+^ GC B cells and PCs within the FM^+^ population upon challenge infection further indicates that the secondary immune response promotes further class-switching and the expansion of class-switched GC B cells and PCs from memory B cells. We further observed that the frequency of FM^+^ GC B cells after 2^nd^ infection is much lower as compared to 1^st^ infection which is consistent with another fate mapping report were antigen plus adjuvant immunizations were used (33). In line with our results, this study demonstrated that memory B cells poorly participate in secondary GC responses.

In our experiments we observed a substantial population of B cells with a GC phenotype during the memory phase in both saline control mice and infected mice. Such a population has been described before using the Aid-CreERT2 system and persisted up to 300 days after immunization with sheep red blood cells while FM^+^ GC B cells were gone at 100 days after immunization with NP-CGG in alum which indicates that complex particulate antigens may promote the differentiation or survival of these cells (14). A recent study demonstrated that such cells persisted even after blocking CD40-CD40 ligand interaction to abolish GC structures (15). Further analysis showed that B cells with a GC phenotype (GL7^+^ CD38^low^) were actually part of the memory response (14, 15). This would also explain why we see an increase of this population after secondary *N. brasiliensis* infection but not in the saline control group. By challenge infection of fate mapping mice we could not determine whether FM^+^ GC B cells after 2^nd^ infection are derived from memory B cells or from persistent and reactivated GC B cells. However, the adoptive transfer experiment indicated that most FM^+^ memory B cells do not participate in GC reactions but rather differentiate to PCs.

The fate mapping approach further allowed us to analyze the memory B cell population within the FM^+^ cells by excluding GC B cells and PCs in the gating strategy. Several studies have shown that triple^+^ and PD-L2^+^ cells are more differentiated and higher affine memory B cells which left the GC reaction late and upon reactivation rapidly differentiate to antibody secreting PCs (14, 15, 37, 38). The triple^+^ population of IgG1 memory B cells has further been shown to give rise to high-affinity IgE-producing PCs while the PD-L2^+^CD73^-^CD80^-^ population contributed to a delayed low-affinity IgE response (28). Transcriptional profiling of IgG1^+^ memory B cell subsets from *N. brasiliensis* infected BALB/c mice further indicated that the triple^+^ cells are immediate precursors of PCs (28). Triple^-^ cells on the other hand were found to leave the GC reaction relatively early and with lower affinity while maintaining the capacity to re-enter the GC reaction during a challenge response. Interestingly, we observed an increase of all three B cell memory subsets in the LNs indicating generation of more memory B cells within the FM^+^ population whereas the number of memory B cells in the BM remained constant. This demonstrates a localized rather than systemic increase of the memory B cell compartment upon challenge infection.

In several previous studies it was observed that triple^-^ cells mainly consisted of IgM^+^ and IgD^+^ cells whereas the other populations contained more class-switched memory B cells (14, 15, 38). In our experiments we observed that IgM^+^ cells constituted 50-60% of the triple^-^ population but only 30-40% of the triple^+^ population in LNs during the memory phase. On the other hand, the triple^+^ population contained about 10% IgG1^+^ cells while only about 3% were IgG1^+^ in the triple^-^ population. The triple^+^ population was the most abundant subset of FM^+^ memory B cells in all organs and this population massively expanded during challenge infection. In the transfer experiment, FM^+^ cells persisted in the recipient mice but did not increase in numbers or participated in the GC reaction. This is in contrast to the strong increase of FM^+^ GC-like B cells observed after challenge infection of the Aid-CreERT2 x tdTomato mice. This discrepancy might be explained by the lack of memory T helper cells that could promote this response or simply by altered responsiveness of B cells due to handling during the adoptive transfer.

Nonetheless, the majority of transferred FM^+^ cells became PCs which shows that the *N. brasiliensis*-specific memory B cells rapidly differentiated to PCs. This is also in line with the current view that memory B cells are part of an immediate defense response by conversion to antibody secreting PCs and only a very small fraction would enter the GC again where they compete with newly developing GC B cells without outcompeting them (19). This scenario would also support the results of the sequencing experiment where we saw no increase of somatic mutations indicating that the transferred FM^+^ cells did not undergo further affinity maturation in GCs but rather immediately differentiated to PCs.

Interestingly, we could show that both BM and LNs were important niches for survival of memory B cells. However, after challenge infection we found that the memory B cells increased in LNs but not in the BM indicating a localized antigen-dependent re-activation. Furthermore, transferred FM^+^ cells derived from either mes-LN or BM both participated in the immune response of the recipient mouse by differentiating to PCs and contributing to the serum IgE levels.

In summary, we could show that induction of Cre activity by a single injection of tamoxifen results in efficient and persistent labelling of B cells that expressed *Aid* at the time of tamoxifen administration during infection with the complex pathogen *N. brasiliensis*. To our knowledge, this is the first GC B cell fate-mapping study in the context of a helminth infection. Using this approach we observed that 80% of GC B cells and 30-50% of PCs in LNs could be labeled during 1^st^ infection. A 2^nd^ infection during the memory phase elicited massive expansion of FM^+^ GC B cells and PCs demonstrating the presence of functional memory B cells. Furthermore, FM^+^ memory B cells in LNs but not in BM increase in numbers and were dominated by triple^+^ cells with relatively low frequencies of IgM^+^ cells. These results provide new insights in the population dynamics of polyclonal B cell responses after helminth infection. This experimental model could further be used to optimize vaccination strategies to increase the number of long-lived plasma cells and support the persistence of memory B cells.

## Data availability statement

The datasets presented in this study can be found in online repositories. The names of the repository/repositories and accession number(s) can be found below: https://www.ncbi.nlm.nih.gov/geo/, GSE209523.

## Ethics statement

The animal study was reviewed and approved by Ethics committee of the Government of Lower Franconia.

## Author contributions

PH and DV designed experiments, PH, and RGG performed experiments, PH, SS, THW and DV analyzed data, PH and DV wrote the manuscript, DV and THW acquired funding.

## Funding

This work was supported by grants from the Deutsche Forschungsgemeinschaft (TRR130_P20 to DV and TRR130_P11 to THW).

## Acknowledgments

We thank Claude-Agnès Reynaud for providing AIDCreERT2 mice, Daniela Döhler, Kirstin Castiglione, Natalie Thuma and Andrea Schneider for technical assistance and members of the Voehringer lab for helpful discussions.

## Conflict of interest

The authors declare that the research was conducted in the absence of any commercial or financial relationships that could be construed as a potential conflict of interest.

## Publisher’s note

All claims expressed in this article are solely those of the authors and do not necessarily represent those of their affiliated organizations, or those of the publisher, the editors and the reviewers. Any product that may be evaluated in this article, or claim that may be made by its manufacturer, is not guaranteed or endorsed by the publisher.

## Abbreviations:

GC, germinal center; PCs, plasma cells; med-LN, mediastinal lymph node; mes-LN, mesenteric lymph node; BM, bone marrow; FM, fate-map; RT, room temperature; BSA, bovine serum albumin; PBS, phosphate buffered saline; FCS, fetal calf serum.
