# Supplementary material for: B cell fate mapping reveals their contribution to the memory immune response against helminths
Source: Front Immunol. 2022 Nov 24;13:1016142. doi: 10.3389/fimmu.2022.1016142 (PMC9730276; doi:10.3389/fimmu.2022.1016142)
Supplement: Supplementary file 1 [file DataSheet_1.docx]

**Supplementary table S1: List of used antibodies and reagents**

| Antibodies | Conjugate | Clone | SOURCE | IDENTIFIER |
| --- | --- | --- | --- | --- |
| Goat Anti-Mouse IgA | AF647 | polyclonal | Southern Biotech | Cat# 1040-31 |
| Goat Anti-Mouse IgE; detection total IgE | AP | polyclonal | Southern Biotech | Cat# 1110-04 |
| Hamster Anti-Mouse CD80 | BV650 | 16-10A1 | BD bioscience | Cat# 563687 |
| Mouse Anti-Mouse CD45.1 | PerCP/Cyanine5.5 | A20 | Thermo Fisher | Cat# A16363 |
| Mouse Anti-Mouse CD45.2 | AF488 | 104 | Biolegend | Cat# 109816 |
| Mouse Anti-Mouse IgE [a]; detection IgE [a] | Biotin | UH297 | Biolegend | Cat# 408804 |
| Mouse Anti-Mouse IgE [b]; detection IgE [b] | Biotin | JKS-6 | Biolegend | Cat# 408704 |
| Mouse IgE κ Isotype Control (clone); [a] allotype standard | Purified | C38-2 | BD bioscience | Cat# 557079 |
| Mouse IgE κ Isotype Control (clone); [b] allotype standard | Purified | C48-2 | BD bioscience | Cat# 557080 |
| Rat Anti-Mouse CD16/32 | Purified | 2.4G2 | Bio x Cell | Cat# BP0307 |
| Rat Anti-Mouse CD19 | PE-Cy7 | eBio(1D3) | Thermo Fisher | Cat# 25-0193-81 |
| Rat Anti-Mouse CD38 | PE-Cy7 | 90 | Biolegend | Cat# 102718 |
| Rat Anti-Mouse CD38 | PerCP/Cyanine5.5 | 90 | Biolegend | Cat# 102722 |
| Rat Anti-Mouse CD45R/B220 | BUV496 | RA3-6B2 | BD bioscience | Cat# 612950 |
| Rat Anti-Mouse CD73 | FITC | TY/11.8 | Biolegend | Cat# 127220 |
| Rat Anti-Mouse CD138 | BV785 | 281-2 | Biolegend | Cat# 142534 |
| Rat Anti-Mouse CD267/TACI | BV421 | 8F10 | BD bioscience | Cat# 742840 |
| Rat Anti-Mouse CD273/PD-L2 | PE-Cy7 | Ty25 | Biolegend | Cat# 107214 |
| Rat Anti-Mouse IgE | BV650 | R35-72 | BD bioscience | Cat# 564208 |
| Rat Anti-Mouse IgE | FITC | R35-72 | BD bioscience | Cat# 553415 |
| Rat Anti-Mouse IgE; Coating | Purified | R35-72 | BD bioscience | Cat# 553413 |
| Rat Anti-Mouse IgG1 | BUV737 | X56 | BD bioscience | Cat# 748403 |
| Rat Anti-Mouse IgM | APC-eFluor 780 | II/41 | Thermo Fisher | Cat# 47-5790-82 |
| Rat Anti-Mouse/Human GL7 Antigen | Biotin | GL7 | Biolegend | Cat# 144616 |
| **Other staining reagents** | Conjugate | Clone | SOURCE | IDENTIFIER |
| eBioscience™ Fixable Viability Dye | eFluor™ 506 | n/a | Thermo Fisher | Cat# 65-0866-14 |
| Streptavidin | AP | n/a | Southern Biotech | Cat# 7105-04 |
| Streptavidin | BUV395 | n/a | BD bioscience | Cat# 564176 |


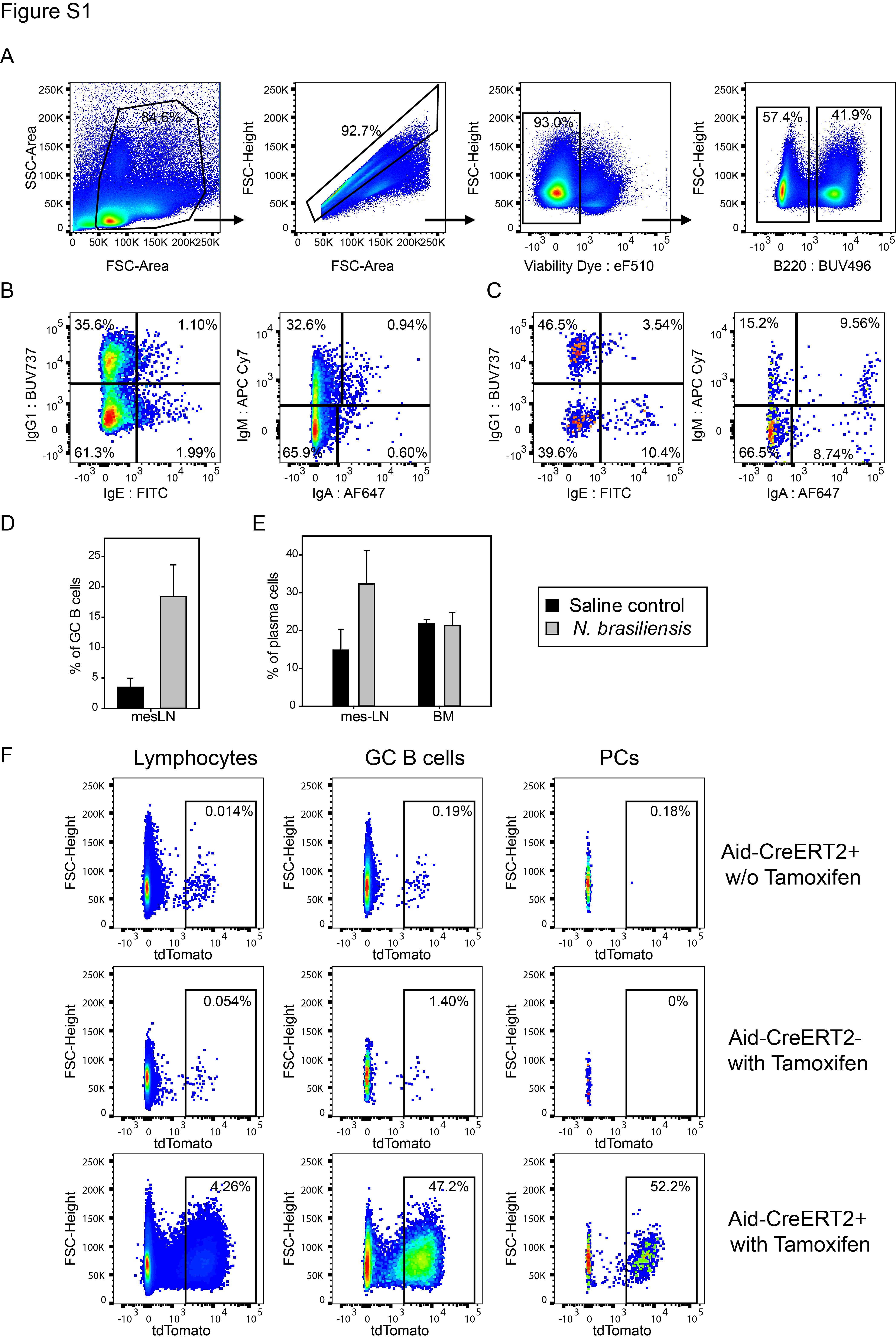


**Figure S1 (related to Fig. 1 and 2). Gating strategy and controls for for GC B cells and PCs.** (**A**) Exemplary gating for B220^+^ and B220^−^ cells for Fig. 1. (**B**, **C**) Exemplary gating for IgG1, IgE, IgM and IgA on GC B cells (**B**) and PCs (**C**) for Fig. 2. Representative plots are from mesenteric lymph nodes of an infected mouse on day 10 after primary *N. brasiliensis* infection. (**D, E**) Comparison % FM^+^ cells on day 10 after challenge infection with 8 months between infections for GC B cells (**D**) and PCs (**E**). (**F**) Representative plots on day 10 after primary *N. brasiliensis* infection for control mice without Cre-recombinase or without Tamoxifen administration.


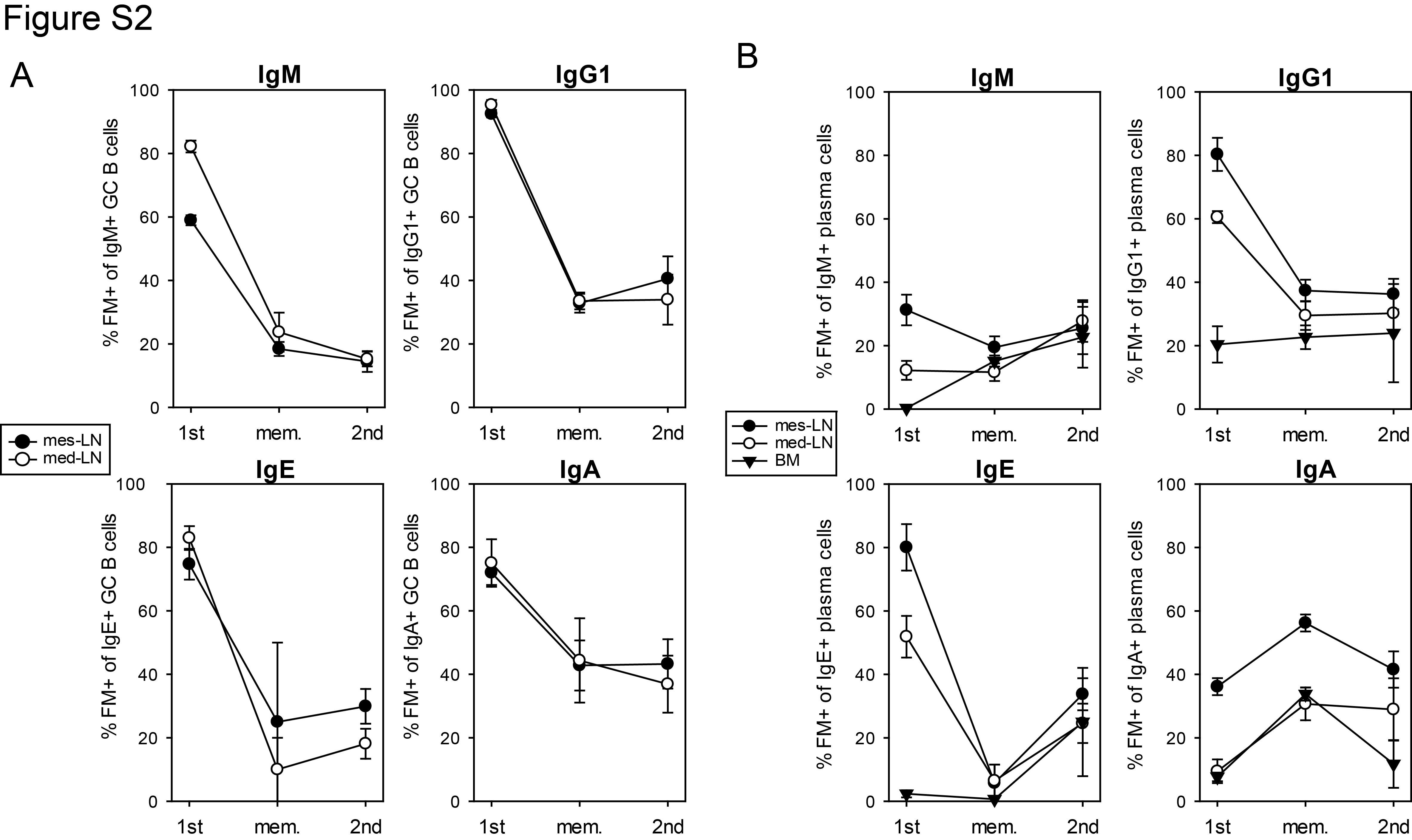


**Figure S2 (related to Fig. 2). FM^+^ cells within Ig isotypes after 1^st^ and 2^nd^ infection.** Percentage of FM^+^ cells within IgM, IgG1, IgE and IgA expressing GC B cells (**A**) or PCs (**B**) on d10 primary infection (1^st^), in the memory phase (mem.) and on d10 after challenge infection (2^nd^) in mes-LN, med-LN or BM. Data shows mean with SEM from 1-3 experiments with 4-8 mice per group.


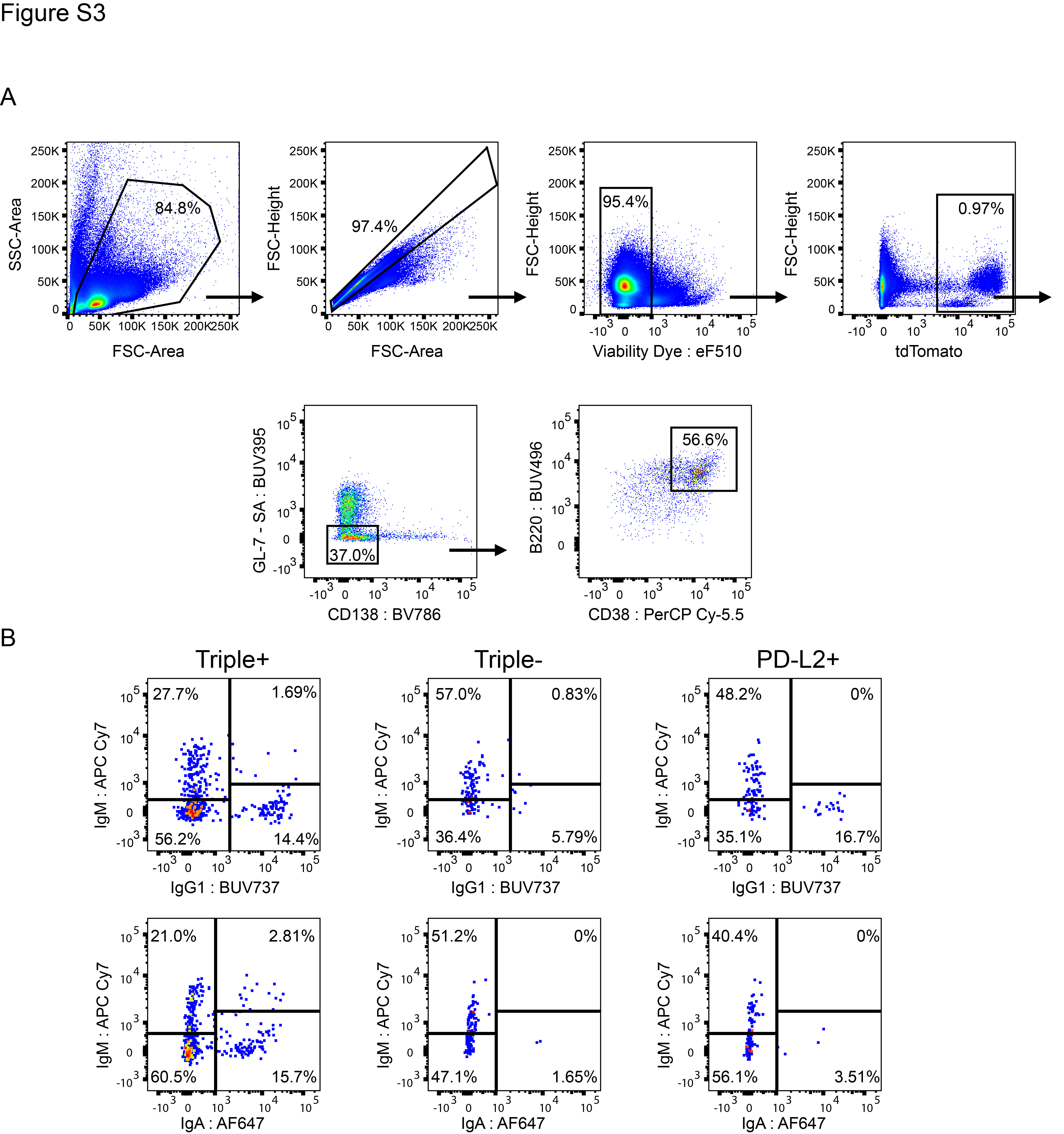


**Figure S3 (related to Fig. 3). Gating strategy for memory B cells.** (**A**) Exemplary gating for memory B cells. (**B**) Exemplary gating for IgA, IgG1 and IgM on memory B cells subsets. Representative plots are from mesenteric lymph nodes of an infected mouse on day 39 after primary *N. brasiliensis* infection.


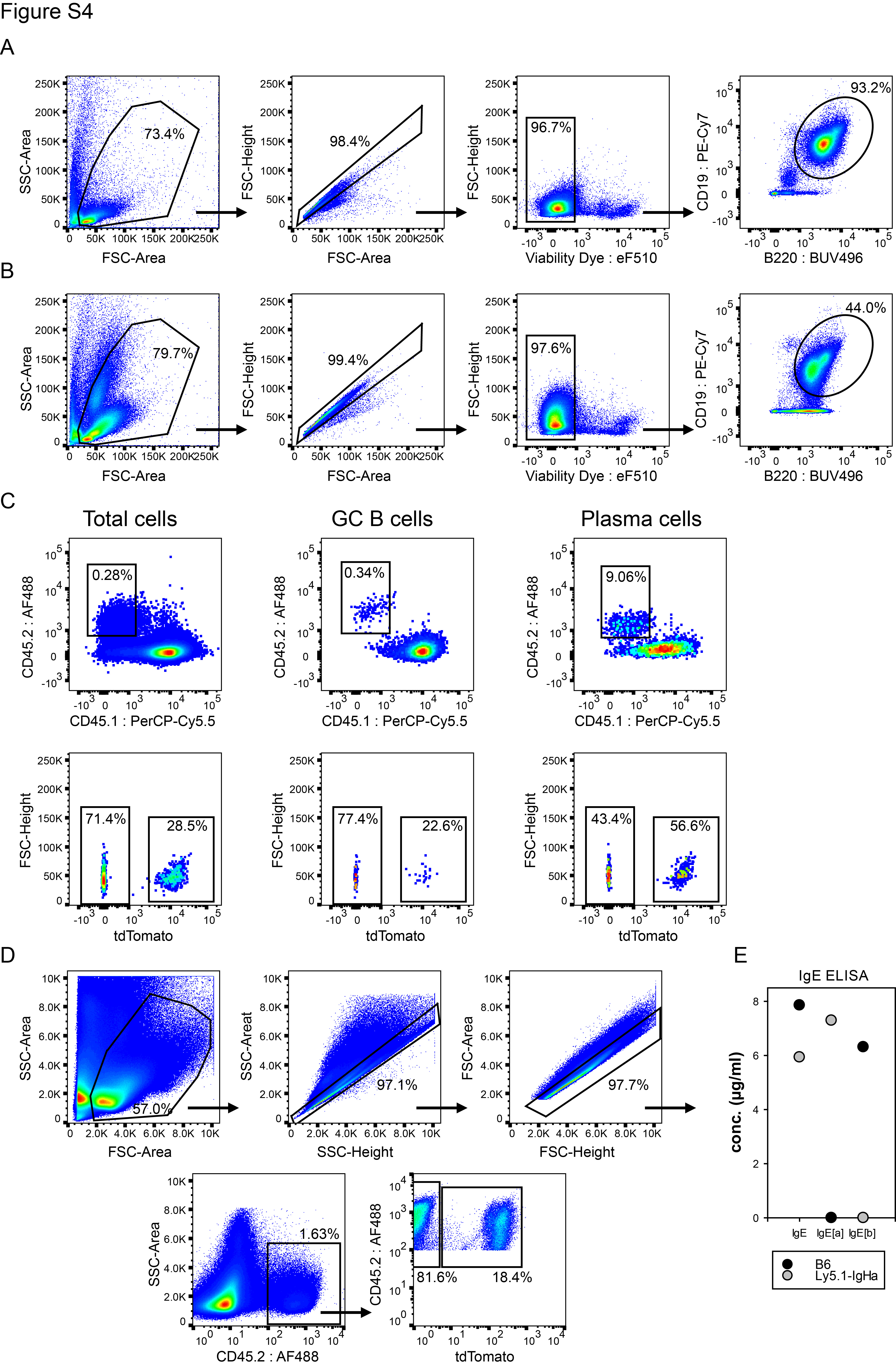


**Figure S4 (related to Fig. 4). Gating strategy for adoptive transfer.** (**A**, **B**) Purity after magnetic cell enrichment from mesenteric lymph nodes (**A**) and bone marrow (**B**). (**C**) Identification of CD45.2^+^FM^+^ (tdTomato^+^) cells after transfer in total cells, GC B cells and plasma cells. (**D**) Exemplary gating for sorting of CD45.2^+^FM^+^ (tdTomato^+^) cells after transfer for sequencing. Representative plots are shown. (**E**) Controls for IgE^a^ and IgE^b^ allotype specific ELISA. Serum from wildtype B6 or Ly5.1-IgH^a^ mouse were collected on day 10 after primary *N. brasiliensis* infection.
